# Polar Value Analysis of Low to Moderate Astigmatism with Wavefront-Guided Sub-Bowman Keratomileusis

**DOI:** 10.1155/2017/5647615

**Published:** 2017-08-02

**Authors:** Pisong Yan, Zhiyu Du, Yu Zhang

**Affiliations:** ^1^Medal Eye Institute, Chongqing, China; ^2^Department of Ophthalmology, Second Affiliated Hospital, Chongqing Medical University, Chongqing, China

## Abstract

**Purpose:**

To evaluate the astigmatic outcomes of wavefront-guided sub-Bowman keratomileusis (WFG-SBK) for low to moderate myopic astigmatism.

**Methods:**

This study enrolled 100 right eyes from 100 patients who underwent WFG-SBK for the correction of myopia and astigmatism. The polar value method was performed with anterior and posterior corneal astigmatism measured with Scheimpflug camera combined with Placido corneal topography (Sirius, CSO) and refractive astigmatism preoperatively and 1 month, 3 months, and 6 months postoperatively.

**Results:**

Similar results for surgically induced astigmatism (SIA) and error of the procedure in both anterior corneal astigmatism (ACA) and total ocular astigmatism (TOA). There was a minor undercorrection of the cylinder in both ACA and TOA. Posterior corneal astigmatism (PCA) showed no significant change.

**Conclusions:**

Wavefront-guided SBK could provide good astigmatic outcomes for the correction of low to moderate myopic astigmatism. The surgical effects were largely attributed to the astigmatic correction of the anterior corneal surface. Posterior corneal astigmatism remained unchanged even after WFG-SBK for myopic astigmatism. Polar value analysis can be used to guide adjustments to the treatment cylinder alongside a nomogram designed to optimize postoperative astigmatic outcomes in myopic WFG-SBK.

## 1. Introduction

Uncorrected astigmatism in most persons with healthy eyes, even when as low as 1.00 diopter (D), can lead to substantial reductions in visual performance [1, 2]. Therefore, in order to achieve better visual performance through refractive surgery, it is crucial to accurately measure ocular astigmatism and precisely treat astigmatism by means of an excimer laser ablation. Moreover, astigmatisms are vectors with defined directions and magnitudes, and the quantitative analysis of astigmatic change might have been eliminated important information and might yield inconsistent results without accounting for the polar nature of astigmatism [3]. The polar value method described by Næser [4] is an excellent way of understanding the precise change in the astigmatic component of refractive surgery. To use this method, some previous studies [5–7] assessed surgically induced astigmatism, error of treatment, and change in the astigmatic component after various refractive surgery. However, to our knowledge, few studies have examined quantitative astigmatic outcomes in wavefront-guided sub-Bowman keratomileusis (WFG-SBK) [8, 9].

The current study was aimed to retrospectively assess quantitative astigmatic outcomes of corneal astigmatism of the anterior and posterior corneal surfaces as well as refractive astigmatism after WFG-SBK for low to moderate myopic astigmatism.

## 2. Materials and Methods

This retrospective observational study comprised 100 right eyes of 100 patients (45 men, 55 women; mean age 23.63 years ± 5.26 [SD], range: 18 to 37 years) who were treated for myopic astigmatism using wavefront-guided sub-Bowman keratomileusis (WFG-SBK). All procedures occurred between June 2012 and September 2015. Only the right eye from each patient was included in the study to avoid the potential bias. The research was carried out according to the Declaration of Helsinki and the ethical standards of the local ethics committee. All patients in the study were healthy individuals who met the standard criteria for refractive surgery. Exclusion criteria were any previous ocular surgery, any corneal diseases, or medical conditions that could impair healing of the ocular surface, and central corneal thickness (CCT) whereby the postoperative thickness would be less than 250 *μ*m below the flap.

### 2.1. Surgical Technique

All surgeries were performed by the same surgeon (ZYD), and all surgical procedures were performed with topical anesthesia. 76 patients underwent flap creation using the 60 kHz IntraLase FS femtosecond laser (Abbott Medical Optics (AMO), Santa Ana, California). The femtosecond laser created a 100 *μ*m thick flap, varying in diameter from 8.2 to 8.5 mm, with a superior hinge. 24 patients underwent flap creation using the Amadeus II microkeratome (Ziemer Group AG, Port, Switzerland). The microkeratome created a 120 *μ*m thick flap with 9 mm diameter. Correction was based on preoperative objective refraction. Target postoperative refraction was +0.25 to +0.50 diopters (D) in all eyes. Stromal ablation was done with the VISX Star S4 IR excimer laser (Abbott Medical Optics (AMO), Santa Ana, California). On the basis of the diopters of myopia, the optical zone was selected from 5.5 mm to 6.5 mm with an up to 8.00 mm blend zone. The average intended ablation depth (AD) was 99.73 ± 27.25 *μ*m (range: 47 to 166 *μ*m). The stromal bed was irrigated with balanced salt solution after the excimer laser ablation to remove any debris, and then the flap was repositioned.

After surgery, patients received ofloxacin 0.3% eye drops 4 times daily and dexamethasone 0.1% eye drops 4 times daily for 1 week. Fuorometholone 0.1% eye drops were applied four times daily for 1 month. Carboxymethyl cellulose eye drops were given 4 times daily for 1 month.

### 2.2. Measurements and Analysis for Astigmatism

Patients were examined preoperatively and 1, 3, and 6 months postoperatively. Uncorrected distance visual acuity and corrected distance visual acuity were recorded, and objective and manifest refraction tests were performed during all follow-up visits.

Anterior and posterior corneal surface measurements were achieved using the Scheimpflug camera combined with Placido corneal topography (Sirius, Costruzione Strumenti Oftalmici (CSO), Florence, Italy, software version 2.5). The 4.0 mm pupil wavefront refraction was obtained using a WaveScan aberrometer (Abbott Medical Optics Inc., software version 3.68). All measurements were performed by the same experienced examiner (PSY) acting in line with the manufacturer's guidelines in all visits.

Anterior corneal astigmatism (ACA) and posterior corneal astigmatism (PCA) were identified as the difference between the anterior or posterior corneal surface power of the steepest (*P*_s_) and flattest (*P*_f_) meridians. For the purpose of this calculation, the axial curvature of the anterior and posterior corneal surface inside a 3.0 mm circular zone centered on the vertex is considered. The measured anterior corneal radii (*r*) are converted into power (*P*) using the formula *P* = (*n* − 1)/*r*, where *n* is the corneal refractive index (1.376). Likewise, the measured posterior corneal radii are converted into power using the formula *P* = (1.336 − 1.376)/*r*. Refractive data were transformed from the vertex to the corneal plane and then further to polar values, as described by Næser [4].

The orientation of corneal astigmatism was defined as against-the-rule (ATR) astigmatism in the case of the steepest corneal meridian between 0 to 30° and 150 to 180°, oblique astigmatism between 30 to 60° and 120 to 150°, and with-the-rule (WTR) astigmatism between 60 and 120°.

As Naeser [4] described, any net astigmatism is fully characterized by two polar values, that is the meridional (AKP) and torsional (AKP + 45) powers, where the former is the power acting along a given meridian Φ (i.e., preoperatively steeper meridian) and the latter is the force twisting the astigmatic direction out of that plane, thereby giving rise to a cylinder rotation. For any given surgical meridian Φ, these polar values are given as

(1) $$\begin{array}{c} AKP=meridional\;power=M\times \text{cos}\left[2\times \left(a-\Phi \right)\right], \end{array}$$

(2) $$\begin{array}{c} AKP+45=torsional\;power=M\times \text{sin}\left[2\times \left(a-\Phi \right)\right]. \end{array}$$

Surgically induced astigmatism (SIA) vector is the amount and axis of astigmatic change caused by surgery, which expressed as polar values (AKP, AKP + 45) are the difference between the postoperative and preoperative polar values. The errors in treatment, AKP_error_ and AKP + 45_error_ were obtained by subtraction of the actual postoperative from the intended keratometry or refraction. A positive AKP_error_ indicates an undercorrection and a negative AKP_error_ an overcorrection. A positive value for AKP + 45_error_ results from an anticlockwise torque, while a clockwise torque is revealed by a negative error. Furthermore, average polar values were converted to net cylinder format by means of the following general equations:

(3) $$\begin{array}{c} M=\sqrt{AKP{\left(\Phi \right)}^{2}+AKP{\left(\Phi +45\right)}^{2}}, \end{array}$$

(4) $$\begin{array}{c} a=\text{arctan}\left[\frac{M-AKP\left(\Phi \right)}{AKP\left(\Phi +45\right)}\right]+\Phi . \end{array}$$

### 2.3. Statistical Analysis

All outcome data were recorded in a Microsoft Excel spreadsheet (Microsoft, Redmond, Washington, USA), and statistical calculations were performed using SPSS software version 23.0 for Windows (SPSS Inc., Chicago, Illinois, USA). Data was described as mean ± standard deviation (SD) and tested for normality using the Kolmogorov-Smirnov test. The repeated measures analysis of variance process of the general linear model in SPSS was used before comparing the preoperative and the postoperative data. Statistics were performed as bivariate analyses of the combined mean polar values with calculation of 2-dimensional confidence ellipses and determination of Hotelling *T*^2^, as previously detailed [10]. All *P* values were two-sided and were determined to be statistically significant when the values were less than 0.05.

## 3. Results

**Figure 1:**
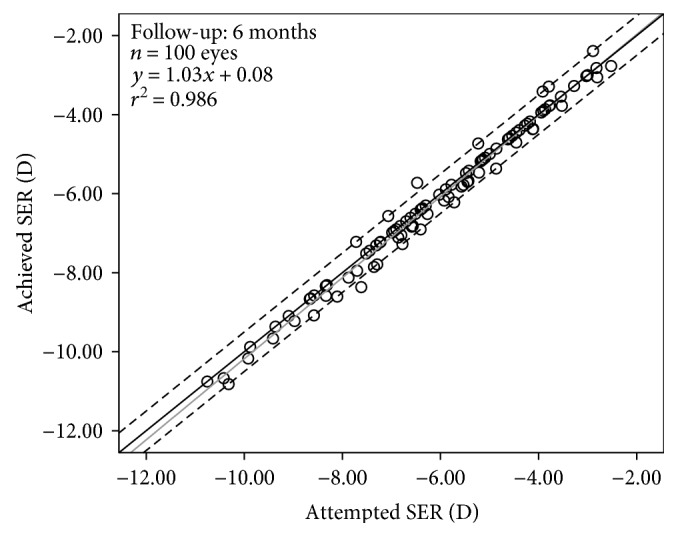
Predictability of spherical equivalent refraction (SER) at 6 months postoperatively. The area between two dotted lines is the postoperative SER within ±0.50 D. The solid grey line represents linear regression.

Mean preoperative subjective sphere and cylinder at corneal plane was −4.74 ± 1.98 D (range: −9.33 to −0.74 D) and −1.16 ± 0.80 D (range: −4.49 to −0.06 D), respectively. Mean preoperative sphere and cylinder by WaveScan at corneal plane was −4.76 ± 2.02 D (range: −9.38 to 0.03 D) and −1.14 ± 0.82 D (range: −4.54 to −0.06 D), respectively. Preoperative and postoperative visual and refractive outcomes were summarized in Table 1. The distributions of preoperative astigmatic components at the corneal plane is shown in Table 2, and 86% of eyes had preoperative astigmatic components at the corneal plane less than or equal to 2.0 D. The spherically equivalent refraction was significantly reduced by 6.20 ± 2.03 D from the preoperative level of −6.12 ± 1.96 D to the postoperative value of 0.08 ± 0.24 D.  Figure 1 shows attempted spherical equivalent refraction (SER) against achieved SER at 6 months postoperatively. The linear regression of the scattergram has a slope of 1.03 and an intercept of 0.08.

### 3.1. Surgically Induced Corneal Astigmatism and Error of Treatment in ACA

**Figure 2:**
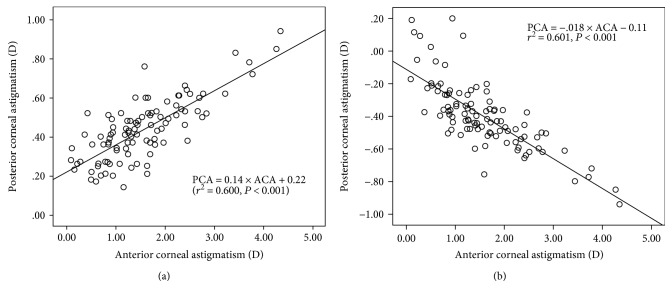
(a) Correlation between the magnitude of anterior corneal astigmatism and posterior corneal astigmatism across the whole sample. (b) Correlation between the meridional polar value AKP of anterior corneal astigmatism and posterior corneal astigmatism across the whole sample. The solid black line represents linear regression.

On the anterior corneal surface, the steeper meridian was vertical in 90 eyes (90.0%), horizontal in 4 eyes (4.0%), and oblique in 6 eyes (6.0%). However, the steepest posterior corneal meridian was vertically aligned in all eyes. The posterior corneal astigmatism was 0.25 D or lower in 12 eyes (12%) and greater than or equal to 0.50 D in 34 eyes (34%). Across the entire sample, a significant correlation was found between the magnitude of PCA and ACA (*P* < .0001, *r* = 0.775, *r*^2^ = 0.600) (Figure 2(a)). The equation that best fits these data was PCA = 0.14 × ACA+ 0.22. A similar correlation was found when ACA and PCA meridional powers were analyzed in relation to the steeper anterior meridian according to (1) (*P* < .0001, *r* = 0.775, *r*^2^ = 0.601) (Figure 2(b)). The equation that best fits these data was PCA = −0.18 × ACA − 0.11. Therefore, ACA was compensated by the PCA in the majority of eyes (94%).

With the steeper meridian of the preoperative TOA as the reference plane, all polar values of ACA before and after surgery are summarized in Table 3. As shown in Table 3, surgery induced a statistically significant flattening of the surgical meridian (*F* = 145.270, *P* < 0.001). There were a minimal induced anticlockwise torque of 0.08 ± 0.39 D, 0.08 ± 0.36 D, and 0.06 ± 0.37 D at 1, 3, and 6 months, respectively. Using (3) and (4) on the polar values, SIA may be expressed as the net cylinder: 0.98 ± 0.56 @ 2.31°, 1.03 ± 0.55 @ 2.24°, and 1.00 ± 0.54 @ 3.95° at 1, 3, and 6 months, respectively. Given the mean intended postoperative astigmatism (target of ACA-AKP), there was a significant undercorrection of astigmatism (or error of treatment) with repeated measures analysis of variance (*F* = 10.176, *P* < 0.001). However, Table 3 shows the relative small mean and the large standard deviations of error of the procedure, indicating a considerable spread with a lot of positive and negative changes. At 6 months, the mean absolute error of meridional power was 0.40 ± 0.29 D and the percentage of eyes with error of meridional power within 0.25 D, 0.50 D, and 1.00 D was 36% (36 eyes), 66% (66 eyes), and 97% (97 eyes), respectively.

**Figure 3:**
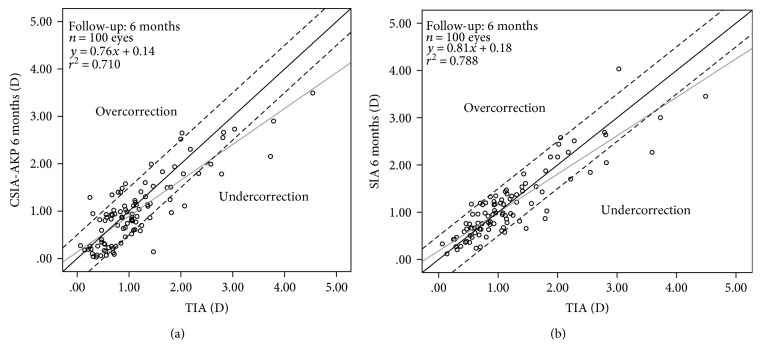
Target-induced astigmatism (TIA) versus surgically induced astigmatism in anterior corneal astigmatism (ACA) (a) and total ocular astigmatism (TOA) (b) at 6 months, expressed as AKP (the astigmatic polar value at the main meridian). Points below the solid black line indicate astigmatic undercorrection. Two dotted lines represent values within ±0.50 D. The solid grey line represents linear regression.

Apart from the changes at the main meridian, there was induced astigmatism at the oblique meridian. Thus, the error of target in the mean torque component (AKP + 45) indicated a minor, but significant, anticlockwise torsion of the cylinder axis (*F* = 3.201, *P* = 0.048). At 6 months, the mean absolute error of torsional power was 0.29 ± 0.23 D and the percentage of eyes with error of torsional power within 0.25 D, 0.50 D, and 1.00 D was 46% (46 eyes), 85% (85 eyes), and 99% (99 eyes), respectively.  Figure 3(a) plots the target-induced astigmatic correction (TIA) versus surgically induced astigmatic correction (SIA) at 6 months, postoperatively. There was a statistically significant correlation between intended and achieved astigmatic correction expressed as AKP (*r*^2^ = 0.710, *P* < 0.001), with an undercorrection of about 24% per diopter (linear regression analysis). The astigmatic error of the treatment in net cylinder notation was calculated to 0.17 @ 10.24° relative to the preoperative stronger meridian at 6 months postoperatively.

For further analysis, eyes in this study were divided into 3 categories based on preoperative TOA: less than or equal to 1.0 D, 1.25 to 2.0 D, and 2.25 D and more.  Table 4 shows polar values and net astigmatism in each category. With the TOA magnitude increase, the higher error of the treatment was found.

**Figure 4:**
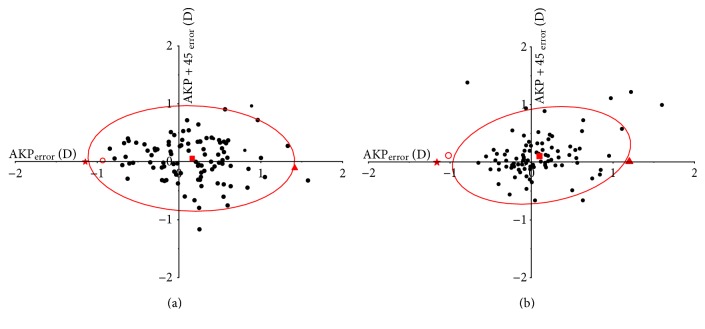
Bivariate analysis of the error of treatment in anterior corneal astigmatism (ACA) (a) and total ocular astigmatism (TOA) (b) following wavefront-guided SBK in 100 eyes. Mean values and 95% confidence areas (solid) of the combined means, expressed as polar values in the preoperatively steeper meridian. The means are shown as follows: preoperative value = triangle to the right; target-induced astigmatism (TIA) = star to the left; surgically induced astigmatism (SIA) = circle to the left; the combined mean of the error of treatment = square in the middle. All values are given in diopters.

Figure 4(a) shows the bivariate analysis of the polar values for dioptric error. Confidence ellipses are given for individual samples and for the combined mean and show a significant combined error of treatment from zero at 6 months, postoperatively (Hotelling's *T*^2^ = 14.669, *P* < 0.01).

With the steeper meridian of the preoperative PCA as the reference plane, mean meridional power in PCA (AKP) was 0.44 ± 0.16 D preoperatively and 0.46 ± 0.37 D, 0.43 ± 0.29 D, and 0.42 ± 0.26 D at 1 month, 3 months, and 6 months postoperatively, respectively. There was no change in the AKP of PCA between the four visits using the repeated measures analysis of variance process (*P* = 0.523). The mean torque component (AKP + 45) in PCA was zero preoperatively and −0.03 ± 0.32 D, −0.02 ± 0.18 D, and 0.01 ± 0.18 D at 1 month, 3 months, and 6 months postoperatively, respectively. No torsion in the AKP + 45 of PCA was found between the four visits (*P* = 0.498).

### 3.2. Surgically Induced Astigmatism and Error of Treatment in TOA

**Figure 5:**
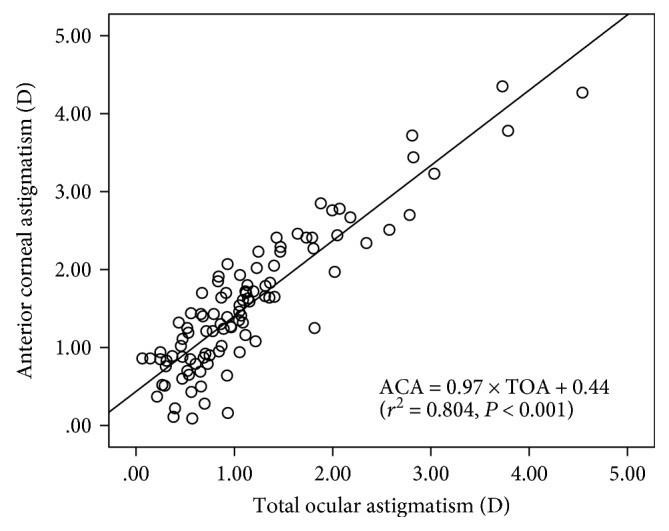
Correlation between the meridional polar value AKP of anterior corneal astigmatism and total ocular astigmatism across the whole sample. The solid black line represents linear regression.

The distribution of preoperative TOA at the corneal plane is shown in Table 2. The TOA was 1.00 D or lower in 53 eyes (53%), exceeded 1.00 D and less than 2.00 D in 33 eyes (33%), and greater than or equal to 2.00 D in 14 eyes (14%). The steeper meridian of TOA was vertical in 78 eyes (78.0%), horizontal in 10 eyes (10.0%), and oblique in 12 eyes (12.0%).  Figure 5 shows a significant correlation between the magnitude of TOA and ACA (*P* < .0001, *r* = 0.897, *r*^2^ = 0.804). The equation that best fits these data was ACA = 0.97 × TOA+ 0.44. The difference in the location of the steep meridian as determined by TOA versus ACA was higher than 15 degrees in 22 eyes (22%).

For further analysis, our series was divided into two groups: group 1, “agreement group” (78 eyes) with a difference location of the steep meridian between TOA and ACA ≤ 15°, and group 2, “disagreement group” (22 eyes) with a difference > 15°. There was a difference in the preoperative TOA (1.32 ± 0.82 D in the agreement group and 0.56 ± 0.29 D in the disagreement group, *P* < .001). No significant difference was found between the 2 groups in the error of treatment at 6 months postoperatively. After matching for preoperative TOA, there was still no difference in the error of treatment between the 2 groups at 6 months postoperatively.

With the steeper meridian of the preoperative TOA as the reference plane, all astigmatic polar values of TOA before and after surgery are summarized in Table 5. As shown in Table 5, surgery induced a statistically significant flattening of the surgical meridian (*F* = 191.357, *P* < 0.001). There was a minimally induced anticlockwise torque of 0.12 ± 0.34 D, 0.13 ± 0.36 D, and 0.10 ± 0.36 D at 1, 3, and 6 months, respectively. Using (3) and (4) on the polar values, SIA may be expressed as the net cylinder: 1.03 ± 0.50 @ 1.23°, 1.04 ± 0.51 @ 1.03°, and 1.06 ± 0.52 @ 1.84° at 1, 3, and 6 months, respectively. Due to zero postoperative astigmatism being targeted in all eyes, the error of treatment is therefore identical to the postoperative astigmatic result in the TOA. The dioptric errors of the procedure at three postoperative visits were examined with repeated measures analysis of variance (*F* = 8.959, *P* < 0.001). Similar to the anterior corneal surface, Table 5 also shows a significant undercorrection of astigmatism. At 6 months, the mean absolute error of meridional power was 0.28 ± 0.29 D and the percentage of eyes with error of meridional power within 0.25 D, 0.50 D, and 1.00 D was 64% (64 eyes), 82% (82 eyes), and 97% (97 eyes), respectively.

Apart from the changes at the main meridian, there was induced astigmatism at the oblique meridian. Thus, the error of target in the mean torque component (AKP + 45) indicated a minor, but significant, anticlockwise torsion of the cylinder axis (*F* = 10.297, *P* < 0.001). At 6 months, the mean absolute error of torsional power was 0.25 ± 0.28 D and the percentage of eyes with error of torsional power within 0.25 D, 0.50 D, and 1.00 D was 68% (68 eyes), 85% (85 eyes), and 96% (96 eyes), respectively.

Figure 3(b) plots the target-induced astigmatic correction (TIA) versus surgically induced astigmatic correction (SIA) at 6 months, postoperatively. There was a statistically significant correlation between intended and achieved astigmatic correction expressed as AKP (*r*^2^ = 0.788, *P* < 0.001), with an undercorrection of 19% per diopter (linear regression analysis). The astigmatic error of the treatment in the net cylinder format was calculated to 0.15 @ 22.69° relative to the preoperative stronger meridian at 6 months postoperatively.

For further analysis, eyes in this study were divided into 3 categories based on preoperative TOA: less than or equal to 1.0 D, 1.25 to 2.0 D, and 2.25 D and more.  Table 4 shows polar values and net astigmatism in each category. With the TOA magnitude increase, the higher error of the treatment was found.

Figure 4(b) shows the bivariate analysis of the polar values for dioptric error. Confidence ellipses are given for individual samples and for the combined mean and show a significant combined error of treatment from zero at 6 months, postoperatively (Hotelling's *T*^2^ = 12.917, *P* = 0.002).

## 4. Discussion

In the current study, a Scheimpflug camera combined with Placido corneal topography (Sirius) was used to measure the curvature of the anterior and posterior cornea. The accuracy of the Sirius has been demonstrated previously [11, 12]. Recent studies [13–19] have reported mean values for posterior corneal astigmatism (PCA) which range from 0.29 to 0.52 D. In our sample, the mean PCA was 0.42 D @ 179.95° and 31% of eyes had astigmatism of more than 0.50 D. This data is similar to those reported previously [13–19]. In our study, there was a significant correlation between the magnitude of PCA and ACA (*P* < .0001, *r* = 0.775, *r*^2^ = 0.600). Meanwhile, a similar correlation was found when ACA and PCA meridional powers were analyzed in relation to the steeper anterior meridian according to (1) (*P* < .0001, *r* = 0.775, *r*^2^ = 0.601). This indicates that the PCA compensated for ACA in the majority of the eyes (94%), similar to Ho et al. [14], Miyake et al. [15], and Qian et al. [20]. They also found that ACA was reduced by the PCA in 91.4% of eyes, in 96.6% of eyes, and in 95.8% of eyes, respectively. This means that PCA can have a significant influence on total corneal astigmatism (TCA) and total ocular astigmatism (TOA). However, as shown in our results, there was no change in the astigmatic polar value of PCA after WFG-SBK surgery. These results are similar to the values obtained by Kamiya et al. in undergoing refractive lenticule extraction (ReLEx) to correct myopic astigmatism [21]. Therefore, the effect of the astigmatic correction was largely attributed to the anterior corneal surface.

Until now, astigmatic surgical correction with excimer lasers is mainly based on preoperative total ocular astigmatism (TOA). By flattening the preoperative steeper meridian, the surgery could achieve to correct astigmatism. However, the astigmatic power is characterized by magnitude in diopters and direction in degrees [3–7, 10, 16]. Therefore, analysis of surgically induced astigmatism (SIA) should be performed with power vector, such as the polar values. The polar value analysis along fixed the steeper meridian of the preoperative TOA for procedures could give much useful information on the result of surgery.

There was a clear trend toward undercorrection of the cylinder in both ACA and TOA (Figure 3). On the anterior corneal surface, the astigmatic error of the treatment in net cylinder notation was calculated to 0.17 @ 10.24° relative to the preoperative stronger meridian at 6 months postoperatively. Likewise, on the total ocular astigmatism, the astigmatic error of the treatment in the net cylinder format was calculated to 0.15 @ 22.69° relative to the preoperative stronger meridian at 6 months postoperatively. These values were lower than those that Ivarsen et al. [5] reported. They found that the astigmatic error in the net cylinder format was calculated to 0.79 @ −7° relative to the preoperative stronger meridian. This difference can be explained by the fact that we enrolled patients with low to moderate astigmatism, whereas the sample of Ivarsen et al. [5] included a large number of eyes with high astigmatism. However, the intended and achieved astigmatic corrections (expressed as AKP) were significantly correlated in both ACA and TOA. Thus, using linear regression analysis, the astigmatic undercorrection amounted to 24% and 19% per diopter in the ACA and TOA, respectively. Similarly, Ivarsen et al. [5] also found that the astigmatic undercorrection amounted to 21% per diopter in the myopic group. Meanwhile, Allan et al. [22] also found more than 20% undercorrection of the cylinder (as shown in Figure 1, top of their study). For this reason, we believe that the same nomogram might be used in our study and the study of Allan et al. [22] according to the manufacturer's guidelines; namely, the treatment programming included a 5% treatment boost, dioptric adjustments in sphere, and no change in astigmatism in all cases.

For further analysis, Table 4 shows the error of treatment in both ACA and TOA stratified by preoperative cylinder at 6 months postoperatively. We found that with the TOA magnitude increase, the higher error of the treatment was found. In the subgroup of eyes with preoperative refractive cylinder more than 2 D, the highest mean absolute error of meridional power was 0.66 ± 0.42 D and 0.63 ± 0.48 D in ACA and TOA at 6 months postoperatively, respectively. Our findings were similar to those of Qian et al. [6] and Schallhorn et al. [8].

On the basis of these data, a 0.2 D correction was added to the attempted cylinder for each 1.0 D increase in preoperative cylinder (Table 6) for developing the nomogram to improve astigmatic outcomes. However, further study is necessary to assess the astigmatic outcomes by using this new nomogram.

Nevertheless, many factors could contribute to the results of astigmatic surgical correction with excimer lasers. Among these factors, it is necessary to consider disagreement between refraction and corneal topography in the steep meridian. We observed that no significant difference was found between the agreement group and the disagreement group in the error of treatment. After matching for preoperative TOA, there was still no difference in the error of treatment between the 2 groups at 6 months postoperatively. This contradicts the study of Bragheeth and Dua [23] where they found that the magnitude error of astigmatic outcomes by LASIK was greater in the disagreement group than in the agreement group (0.16 ± 0.74 versus 0.07 ± 0.52). The difference was statistically significant between the agreement group and the disagreement group. This difference can be explained by different ablation profiles during surgery. In our study, we performed WFG-SBK, whereas the sample of Bragheeth and Dua [23] used conventional LASIK-corrected myopic astigmatism.

This study has limitations, and further studies are warranted. First, anterior corneal astigmatism was centered on the corneal vertex and not on the pupil center. However, pupil-centered measurements may be more accurate and have to be assessed. Thus, we have started another study to assess astigmatic outcomes after WFG-SBK by using pupil-centered measurements on the anterior corneal surface. Second, the number of patients, especially with oblique astigmatism and ATR astigmatism, was small. Third, the time of follow-up is too short in the study. Since the unpredictable nature of corneal wound healing and the biomechanical response to surgery [24] are present, further study is necessary to assess the refractive change over a longer period of time.

In conclusion, our study confirms that WFG-SBK could provide good astigmatic outcomes for the correction of myopic astigmatism. We observed similar results for SIA and error of the procedure in both ACA and TOA. This means that we can objectively evaluate the astigmatic outcomes for the correction of myopic astigmatism by measuring the changes of ACA before and after operation. Although slight undercorrection (less than a quarter of a diopter) was seen in our dataset, the clinical refractive results are still highly satisfactory. There was no change in the astigmatic polar value of PCA after WFG-SBK surgery. Polar value analysis can be used to guide adjustments to the treatment cylinder alongside the nomogram designed to optimize postoperative astigmatic outcomes in myopic WFG-SBK. Despite some flaws, the current study is one of the few studies reporting results of WFG-SBK in patients with low to moderate refractive cylinder.

## Figures and Tables

**Table 1:** Refractive and visual outcomes (*n* = 100 eyes).

Parameter	Preop mean (SD)	Postop 6 m mean (SD)	*P* value
Sphere (D) (corneal plane)	−4.76 (2.02)	0.53 (0.28)	<0.001
Cylinder (D) (corneal plane)	−1.14 (0.82)	−0.42 (0.36)	<0.001
SER (D) (corneal plane)	−6.12 (1.96)	0.08 (0.24)	<0.001
UDVA (logMAR)	1.16 (0.33)	−0.10 (0.06)	<0.001
CDVA (logMAR)	−0.07 (0.05)	−0.11 (0.07)	>0.05

Note: sphere and cylinder by WaveScan at corneal plane. SER = sphere +1/2^∗^cylinder. Preop: preoperative visit; Postop 6 m: 6 months postoperative visit after surgery; SD: standard deviation; D: diopter; SER: spherical equivalent refraction; UDVA: uncorrected distance visual acuity; CDVA: corrected distance visual acuity.

**Table 2:** Distribution of preoperative astigmatic components (corneal plane, *N* = 100 eyes).

Parameter	Magnitude (D)		Percentage of eyes		
	Mean (SD)	Median (range)	≤1.0 D	1.0 to 2.0 D	≥2.0 D
TOA	1.14 (0.82)	0.93 (0.06 to 4.54)	53	33	14
ACA	1.54 (0.88)	1.41 (0.09 to 4.35)	31	44	25
PCA	−0.44 (0.16)	−0.43 (−0.14 to −0.94)	100	0	0

TOA: total ocular astigmatism, ACA: anterior corneal astigmatism, PCA: posterior corneal astigmatism, D: diopter.

**Table 3:** Polar values in anterior corneal astigmatism (ACA) by Sirius. All measurements with the steeper meridian of the preoperative total ocular astigmatism (TOA) as the reference plane.

	AKP	AKP + 45	Net astigmatism
Preop	1.41 ± 0.98	−0.11 ± 0.43	1.42 ± 0.65 @ 92.52°
Postop 1 m	0.45 ± 0.68	−0.03 ± 0.48	0.45 ± 0.57 @ 93.08°
Postop 3 m	0.44 ± 0.62	−0.03 ± 0.48	0.44 ± 0.55 @ 92.82°
Postop 6 m	0.43 ± 0.60	−0.05 ± 0.49	0.43 ± 0.54 @ 91.60°
*F* value	145.270	3.201	**—**
*P* value	< 0.001	0.048	**—**
SIA 1 m	−0.97 ± 0.79	0.08 ± 0.39	−0.98 ± 0.56 @ 92.31°
SIA 3 m	−0.98 ± 0.77	0.08 ± 0.36	−1.03 ± 0.55 @ 92.24°
SIA 6 m	−0.98 ± 0.77	0.06 ± 0.37	−1.00 ± 0.54 @ 93.95°
TIA	−1.14 ± 0.82	0.00	−1.14 ± 0.82 @ 94.68°
*F* value	11.405	3.201	**—**
*P* value	<0.001	0.048	**—**
Target of ACA	0.27 ± 0.46	−0.11 ± 0.43	0.29 ± 0.45 @ 83.90°
*Error of treatment*			
1 m	0.18 ± 0.49	0.08 ± 0.39	0.19 ± 0.44 @ 107.21°
3 m	0.17 ± 0.48	0.08 ± 0.36	0.18 ± 0.43 @ 107.27°
6 m	0.16 ± 0.47	0.06 ± 0.37	0.17 ± 0.42 @ 104.92°
*F* value	10.176	3.201	**—**
*P* value	<0.001	0.048	**—**

SIA: surgical induced astigmatism, TIA: target-induced astigmatism, ACA: anterior corneal astigmatism.

**Table 4:** Polar value analysis of changes in anterior corneal astigmatism (ACA) and total ocular astigmatism (TOA) stratified by the magnitude of preoperative cylinder at 6 months postoperatively.

	Parameter	≤1.0 D (*n* = 53)	1.0 to 2.0 D (*n* = 34)	>2.0 D (*n* = 13)	*P* value
*Anterior corneal astigmatism*					
Preop	AKP	0.80 ± 0.63	1.75 ± 0.51	3.05 ± 0.77	<0.001
	AKP + 45	−0.06 ± 0.40	−0.12 ± 0.47	−0.25 ± 0.45	0.371
	Net astigmatism	0.80 @ −2.14°	1.75 @ −1.96°	3.06 @ −2.34°	—
Postop 6 m	AKP_error_	0.07 ± 0.40	0.15 ± 0.47	0.55 ± 0.57	0.004
	AKP + 45_error_	0.05 ± 0.34	0.01 ± 0.39	0.21 ± 0.42	0.267
	│AKP_error_│	0.33 ± 0.23	0.40 ± 0.28	0.66 ± 0.42	0.001
	Net astigmatism	0.09 @ 17.77°	0.15 @ 1.91°	0.59 @ 10.45°	—
*Total ocular astigmatism*					
Preop	AKP	0.63 ± 0.23	1.34 ± 0.28	2.81 ± 0.74	<0.001
	AKP + 45	0.00	0.00	0.00	—
	Net astigmatism	0.63 @ 0	1.34 @ 0	2.81 @ 0	—
Postop 6 m	AKP_error_	0.004 ± 0.25	0.16 ± 0.35	0.36 ± 0.72	0.007
	AKP + 45_error_	0.08 ± 0.24	0.002 ± 0.31	0.48 ± 0.62	<0.001
	│AKP_error_│	0.19 ± 0.16	0.30 ± 0.24	0.63 ± 0.48	<0.001
	Net astigmatism	0.08 @ 43.57°	0.16 @ 0.36°	0.60 @ 26.57°	—

**Table 5:** Polar values in manifest refraction by WaveScan. All measurements with the steeper meridian of the preoperative total ocular astigmatism (TOA) as the reference plane.

	AKP	AKP + 45	Net astigmatism
Preop	1.14 ± 0.82	0.00	1.14 ± 0.82 @ 94.68°
Postop 1 m	0.14 ± 0.40	0.12 ± 0.34	0.18 ± 0.37 @ 115.73°
Postop 3 m	0.13 ± 0.38	0.13 ± 0.36	0.18 ± 0.37 @ 117.86°
Postop 6 m	0.10 ± 0.39	0.10 ± 0.36	0.15 ± 0.38 @ 117.37°
*F* value	191.357	10.297	**—**
*P* value	<0.001	<0.001	**—**
SIA 1 m	−1.02 ± 0.72	0.12 ± 0.34	−1.03 ± 0.50 @ 91.23°
SIA 3 m	−1.03 ± 0.74	0.13 ± 0.36	−1.04 ± 0.51 @ 91.03°
SIA 6 m	−1.05 ± 0.74	0.10 ± 0.36	−1.06 ± 0.52 @ 91.84°
TIA	−1.14 ± 0.82	0.00	−1.14 ± 0.82 @ 94.68°
*F* value	8.959	10.297	**—**
*P* value	0.001	<0.001	**—**
*Error of treatment*			
1 m	0.14 ± 0.40	0.12 ± 0.34	0.18 ± 0.37 @ 115.73°
3 m	0.13 ± 0.38	0.13 ± 0.36	0.18 ± 0.37 @ 117.86°
6 m	0.10 ± 0.39	0.10 ± 0.36	0.15 ± 0.38 @ 117.37°
*F* value	8.959	10.297	**—**
*P* value	0.001	<0.001	**—**

**Table 6:** Astigmatism adjustment applied in nomogram according to the manufacturer's guidelines.

Preop manifest refraction cylinder (D)	Addition to treatment cylinder (D)
0.00 to 0.99	0.20
1.00 to 1.99	0.40
2.00 to 2.99	0.60
3.00 to 3.99	0.80
4.00 to 4.99	1.00
